# Supplementary material for: A transcriptional metastatic signature predicts survival in clear cell renal cell carcinoma
Source: Nat Commun. 2022 Sep 30;13:5747. doi: 10.1038/s41467-022-33375-w (PMC9525645; doi:10.1038/s41467-022-33375-w)
Supplement: Supplementary file 1 — Supplementary Figures 1-9 [file 41467_2022_33375_MOESM1_ESM.pdf]

Supplementary Information File

A transcriptional metastatic signature predicts survival in clear cell renal cell carcinoma

Adele M. Alchahin[*], Shenglin Mei[* §], Ioanna Tsea, Taghreed Hirz, Youmna Kfoury, Douglas Dahl, Chin-Lee Wu, Alexander O. Subtelny, Shulin Wu, David T. Scadden, John H. Shin, Philip J. Saylor[†], David B. Sykes[†], Peter V. Kharchenko[†,§],Ninib Baryawno[†,§]

Content: Supplementary Figure 1-9

Supplementary Figure 1

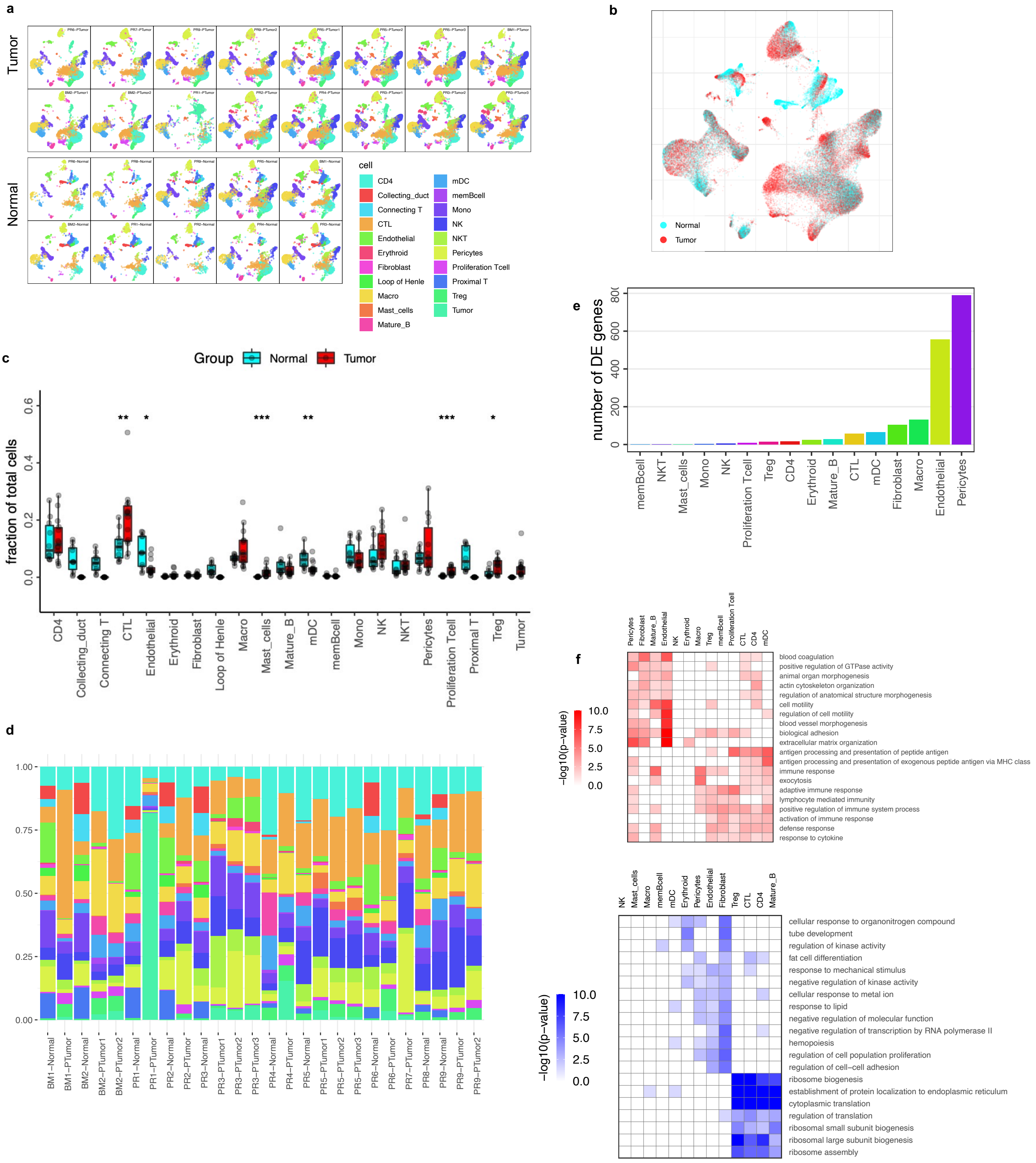

Supplementary Fig. 1: Landscape of tumor microenvironment in primary ccRCC and adjacent normal tissue. **a** UMAP plot showing major cell annotation in individual sample of adjacent normal and primary ccRCC tissue. **b** UMAP visualization of normal kidney tissue and primary RCC cells. **c** Comparison of relative cell abundance of major cell clusters between normal kidney tissue and primary RCC tumor. Statistics are accessed with a two side Wilcoxon rank sum test (CTL ***p*=0.0073; endothelial **p*=0.013; mast cells ****p*=0.00025; mDC ***p*=0.0089; proliferation T cells ****p*=0.00037; Treg **p*=0.011). Boxplots: centercentre line: median; box limits: upper and lower quartiles; whiskers extend at most 1.5x interquartile range past upper and lower quartiles. IQR range between 25th and 75th percentile with mid-point of the data. Single cell samples, tumor n=16 samples, normal adjacent kidney, n=10 samples. **d** Barplot representing the fraction of major cell types within each sample. **e** Number of differentially expressed genes comparing RCC tumor and adjacent normal kidney tissue (cutoff: adjusted *p* value<0.001). **f** GO BP categories with most significant enrichment for the top 300 up- (top) and down-regulated (bottom) genes. The statistical analysis was performed by over-representation test. Source data are provided as a Source Data file.

Supplementary Figure 2

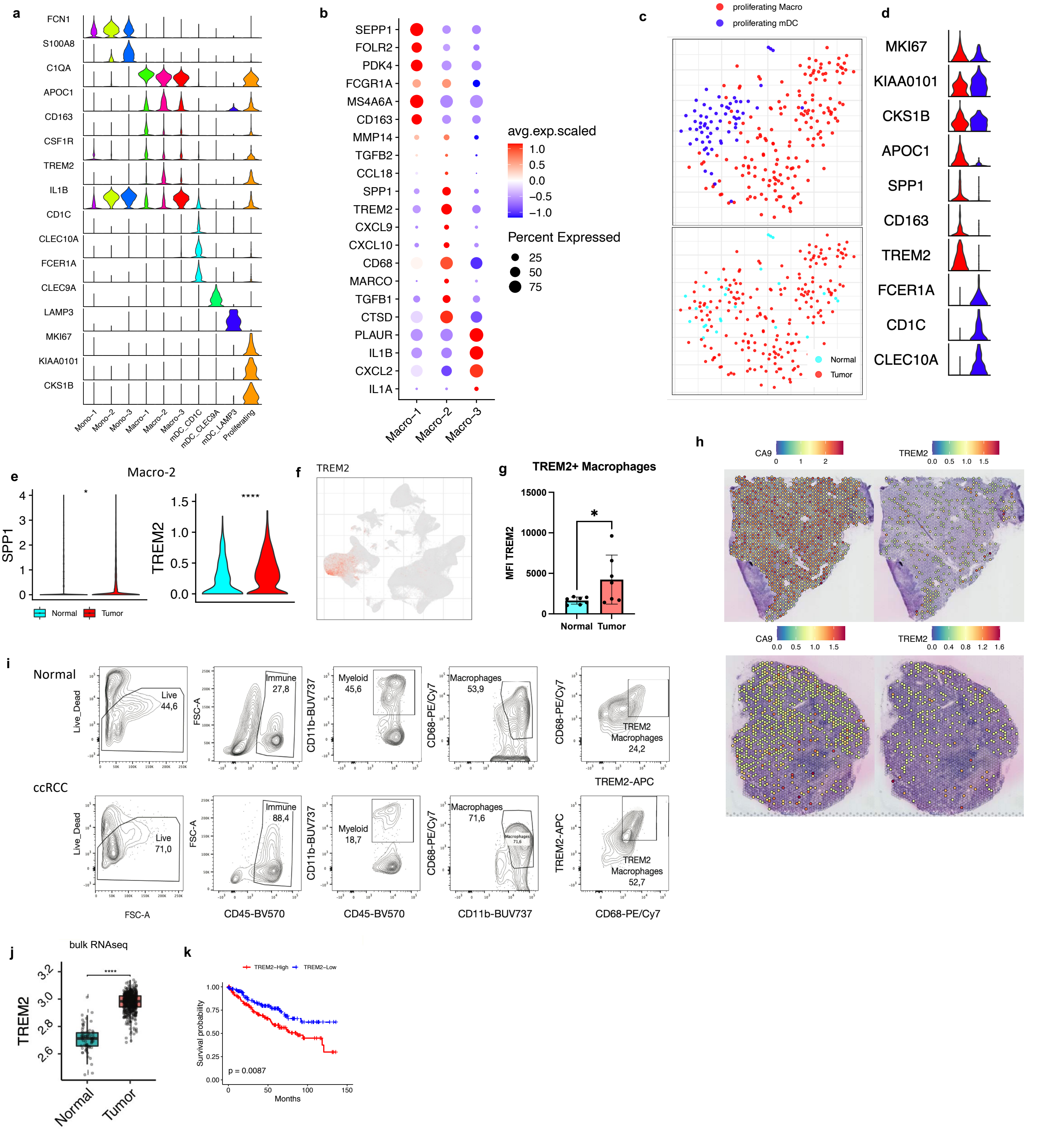

Supplementary Fig. 2: Immunosuppressive macrophages are highly enriched in ccRCC tumors. **a** Violin plot showing representative marker gene expression of myeloid subpopulations. **b** Dot plot showing maker gene expression of macrophage subsets. The colour represents scaled average expression of marker genes in each cell type, and the size indicates the proportion of cells expressing marker genes. **c** Enlarged view of proliferating myeloid cells on UMAP embedding, coloured by cell annotation (left) and sample group. **d** Violin plot showing marker gene expression in proliferating macrophage and proliferating mDC. **e** *SPP1* and *TREM2* expression in Macro-2 shown as Violin plot. **f** UMAP visualization of *TREM2* expression in major cell populations (Figure 1b). **g** Flow cytometric analysis of *TREM2* expression on Macrophages from Tumor and paired adjacent normal tissue. (n= 4 per group). Statistics are accessed with paired two tailed t-test. (*p= 0,0451, error bars: SEM). **h** Spatial feature plots showing *CA9* and *TREM2* expression in ccRCC patient. Tumor spots are marked by *CA9* expression. **i** Gating strategy for flow validation of *TREM2*+ macrophages. Labels above the flow plots refer to the parent population and the percentages are of parent gate (right). **j** *TREM2* expression in TCGA KIRC data is shown as boxplot. Statistics are accessed using a two side Wilcoxon rank sum test (p<2e-16). Boxplots: center line: median; box limits: upper and lower quartiles; whiskers extend at most 1.5x interquartile range past upper and lower quartiles. **k** Kaplan–Meier curves showing higher *TREM2* expression is associated with worse patient survival for TCGA KIRC data (n= 533). Source data are provided as a Source Data file.

Supplementary Figure 3

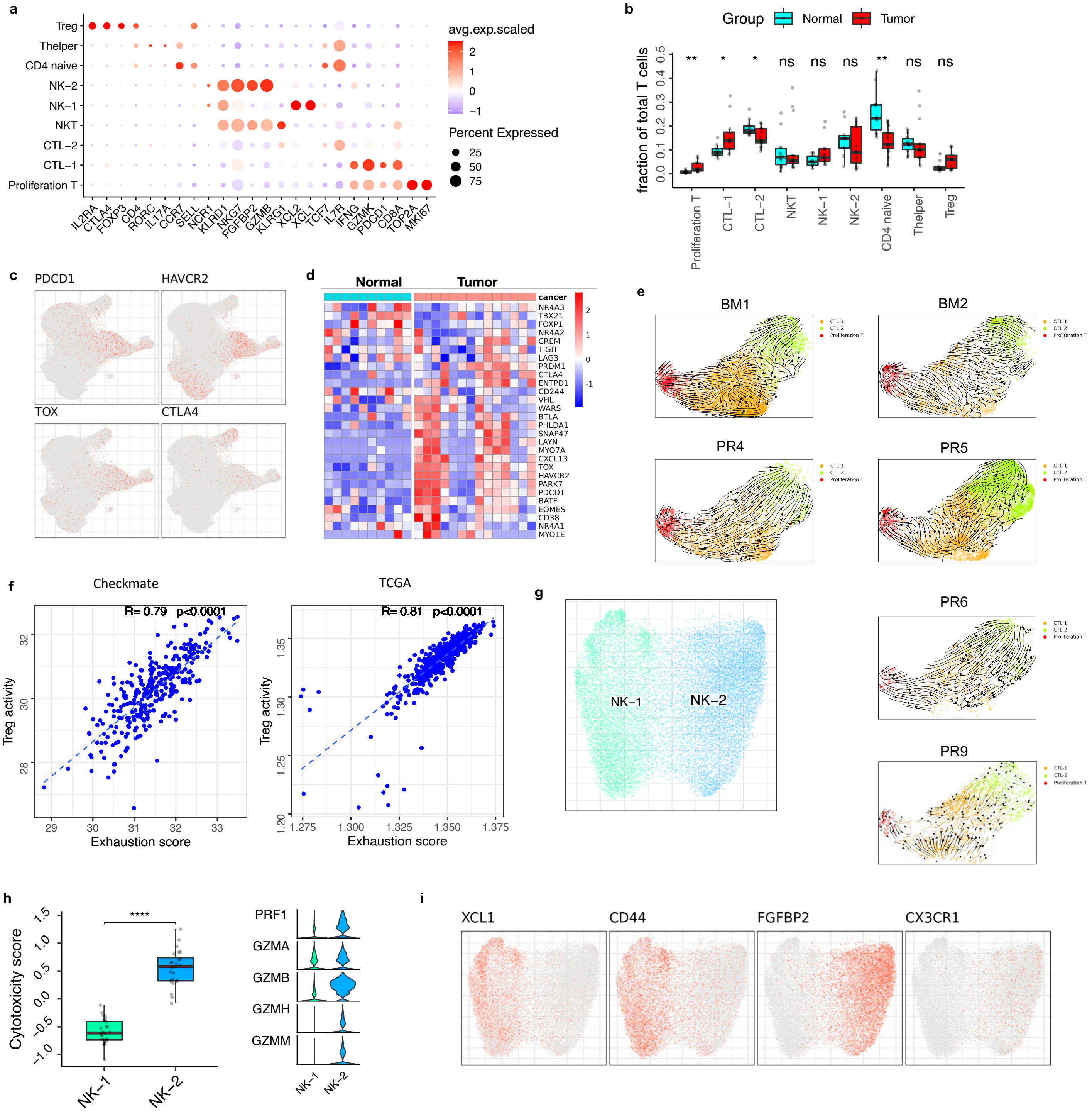

Supplementary Fig. 3: Proliferating T cells may act as progenitors of exhausted T cells in the immunosuppressive T cell environment. **a** Dot plot showing maker gene expression of major cell types. The colour represents scaled average expression of marker genes in each cell type, and the size indicates the proportion of cells expressing marker genes. **b** Comparison of relative cell abundance of different cell clusters between primary RCC and adjacent normal. Significance was assessed using two side Wilcoxon rank sum test (Proliferation T, ***p*=0.0031; CTL-1, ***p*=0.0059; CTL-2, **p*=0,013; CD4 naïve, ***p*= 0,0015). Boxplots: centre line: median; box limits: upper and lower quartiles; whiskers extend at most 1.5x interquartile range past upper and lower quartiles. Single cell samples, tumor n=14 samples, normal adjacent kidney, n=10 samples. **c** Expression of marker genes for the known annotated exhaustion genes visualized on UMAP embedding. **d** Heatmap demonstrating exhaustion signature gene expression in CTL-1. **e** RNA velocity analysis of the transition state in the region of CTL-1, CTL-2 and Proliferating T cells, estimated on individual samples. **f** Correlation of exhaustion signature score and Treg activity score is shown as scatter plot for CheckMate (left) and TCGA KIRC (right) ccRCC bulk RNAseq data. **g** UMAP visualization of NK cell subpopulations. **h** Boxplot presenting the cytotoxicity score of NK-1 and NK2 (left) (*****p*= 6,2 e-14) with a two side Wilcoxon rank sum test. Boxplot represents IQR range between 25th and 75th percentile with mid-point of the data, whiskers indicate the upper and lower value within 1.5times the IQR. Single cell samples, tumor n=14 samples, normal adjacent kidney, n=10 samples. Violin plot showing the expression level of cytotoxicity genes in NK-1 and NK-2. **i** Representative marker gene expression on NK cell subsets. Source data are provided as a Source Data file.

Supplementary Figure 4

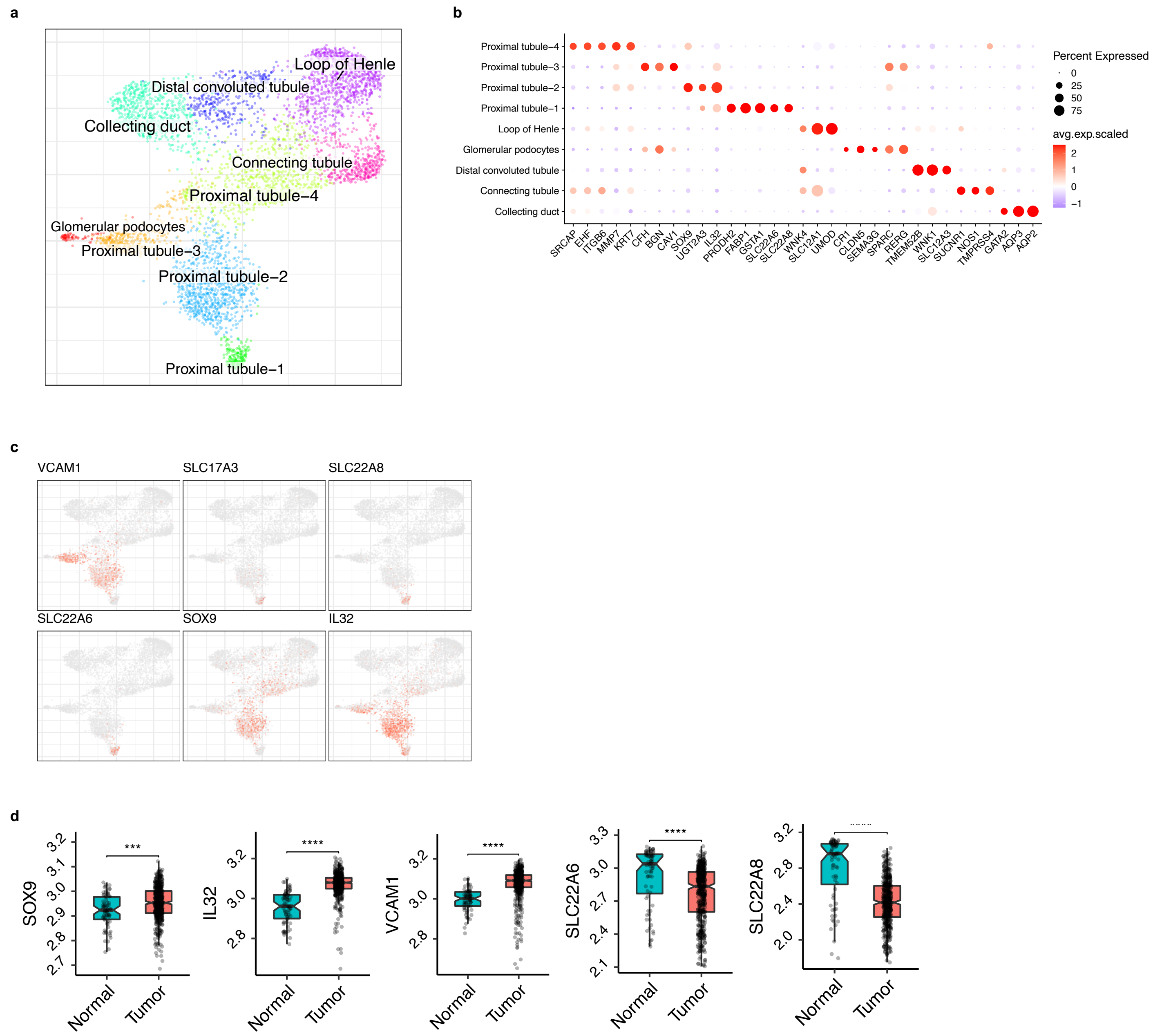

Supplementary Fig. 4: Characterization of malignant cells and sub-clusters. a Annotation of nephron anatomy in normal kidney tissue. **b** Dot plots showing representative marker genes across different subsets in normal kidney tissue. The colour represents scaled average expression of marker genes in each cell type, and the size indicates the proportion of cells expressing marker genes. **c** Expression of representative genes that are upregulated in proximal tubule visualized in a UMAP embedding. **d** Boxplot showing proximal tubule feature gene expression in TCGA KIRC data. Statistics are accessed using a two side Wilcoxon rank sum test. *SOX9* ****p*=0,00029; *IL32* *****p*<2e-16; *VCAM1* *****p* <2e-16; *SLC22A6* *****p*=1,8e-09; *SLC22A8* *****p* <2e-16. Boxplot includes center line: median; box limits: upper and lower quartiles; whiskers extend at most 1.5x interquartile range past upper and lower quartiles. Sample size: Tumor n=533, Normal n=53. Source data are provided as a Source Data file.

Supplementary Figure 5

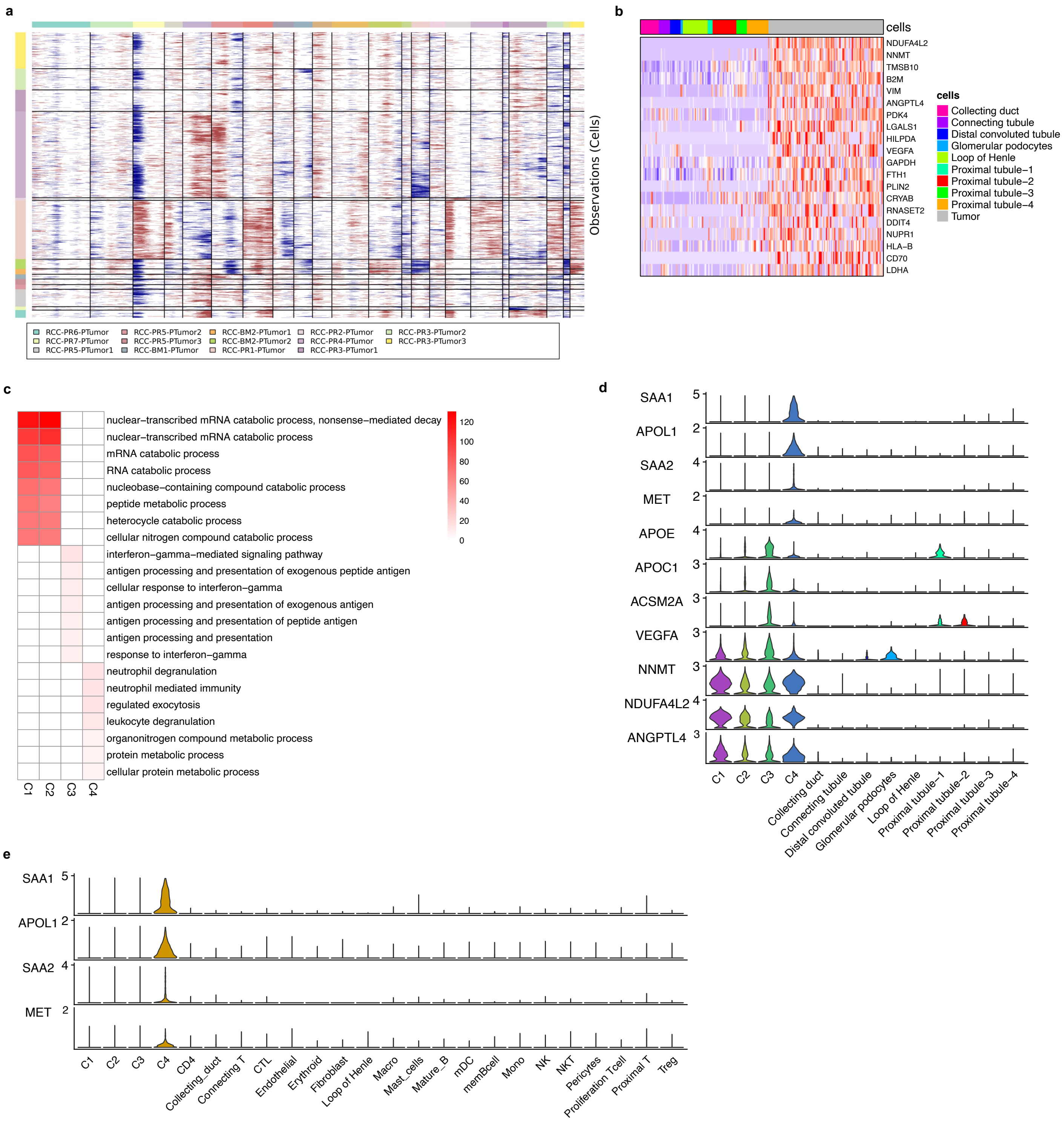

Supplementary Fig. 5: Characterization of malignant cells and sub-clusters. a Inferred CNV profile of tumor cells, grouped by individual patient samples. **b** Heatmap shows an overview of genes (vertical rows) differentially expressed between tumor cells from primary RCC tissue and nephron anatomy from adjacent normal kidney tissue. **c** Overview of enriched GO terms for top 200 upregulated genes in tumor cell sub-clones. The statistical analysis was performed by over-representation test. **d** Violin plot showing genes in different subpopulations of the kidney nephron and the tumor. **e** Violin plot showing tumor metastatic signature gene expression in major cell populations. Source data are provided as a Source Data file.

Supplementary Figure 6

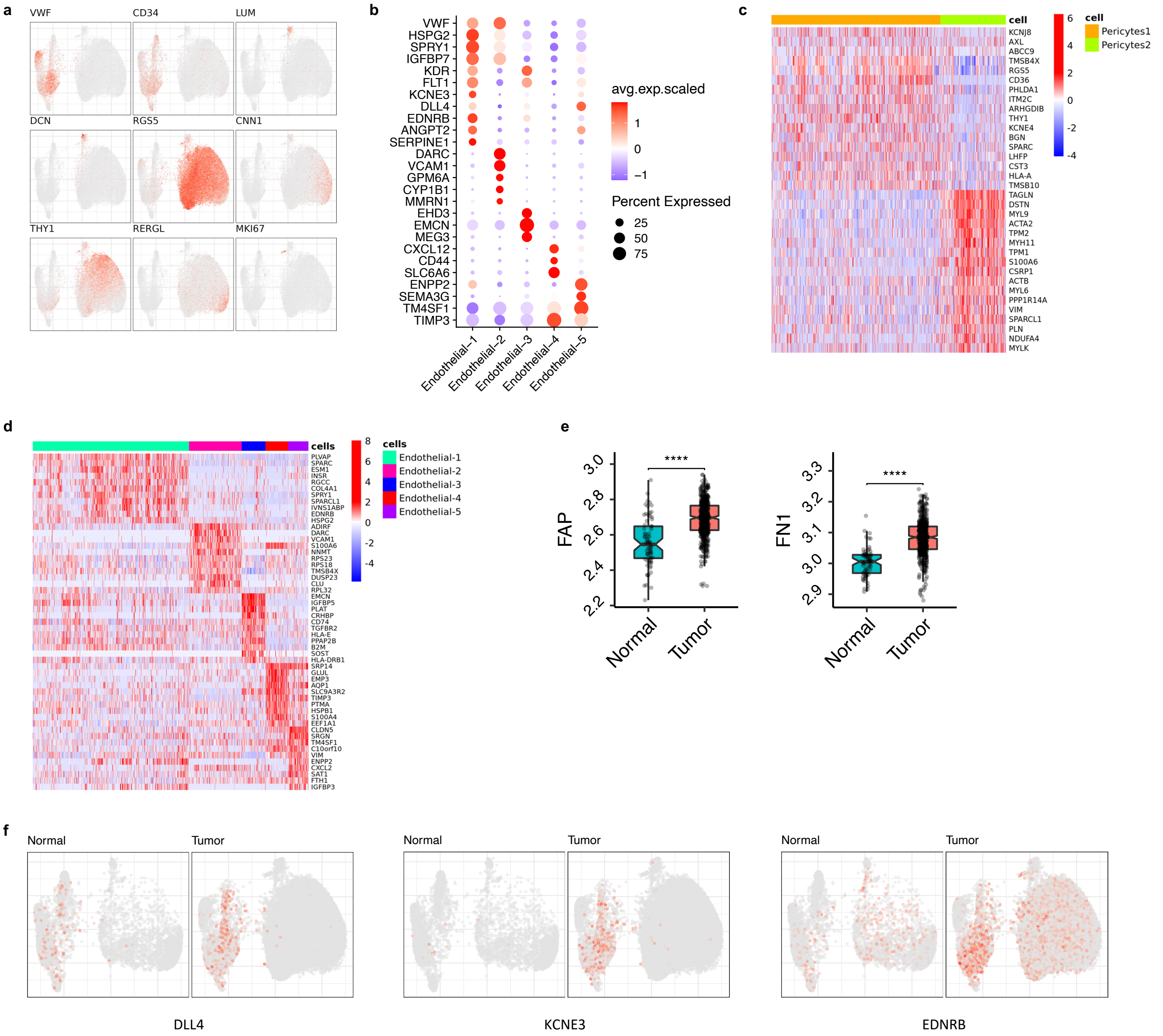

Supplementary Fig. 6: Characterization of stromal subpopulations. a Expression of representative genes in pericytes, endothelial and fibroblasts visualized in a UMAP embedding. **b** Dot plots showing representative marker genes across the different endothelial subsets. The colour represents scaled average expression of marker genes in each cell type, and the size indicates the proportion of cells expressing marker genes. **c** Heatmap shows an overview of genes (vertical rows) differentially expressed between Pericyte-1 and Pericyte-2. **d** Heatmap showing the differential expression of the different endothelial subpopulations. **e** *FN1* and *FAP* expression in TCGA KIRC data are shown as boxplot. Statistics are accessed with a two side Wilcoxon rank sum test. Boxplots: centre line: median; box limits: upper and lower quartiles; whiskers extend at most 1.5x interquartile range past upper and lower quartiles. *FN1* ****p <2e-16; *FAP* ****p=1,4e-15. (Sample size: Tumor n=533, Normal n=53) **f** Expression of *DLL4*, *KCNE3* and *EDNRB* in UMAP embedding comparing adjacent normal kidney tissue and primary ccRCC tumor tissue. Source data are provided as a Source Data file.

Supplementary Figure 7

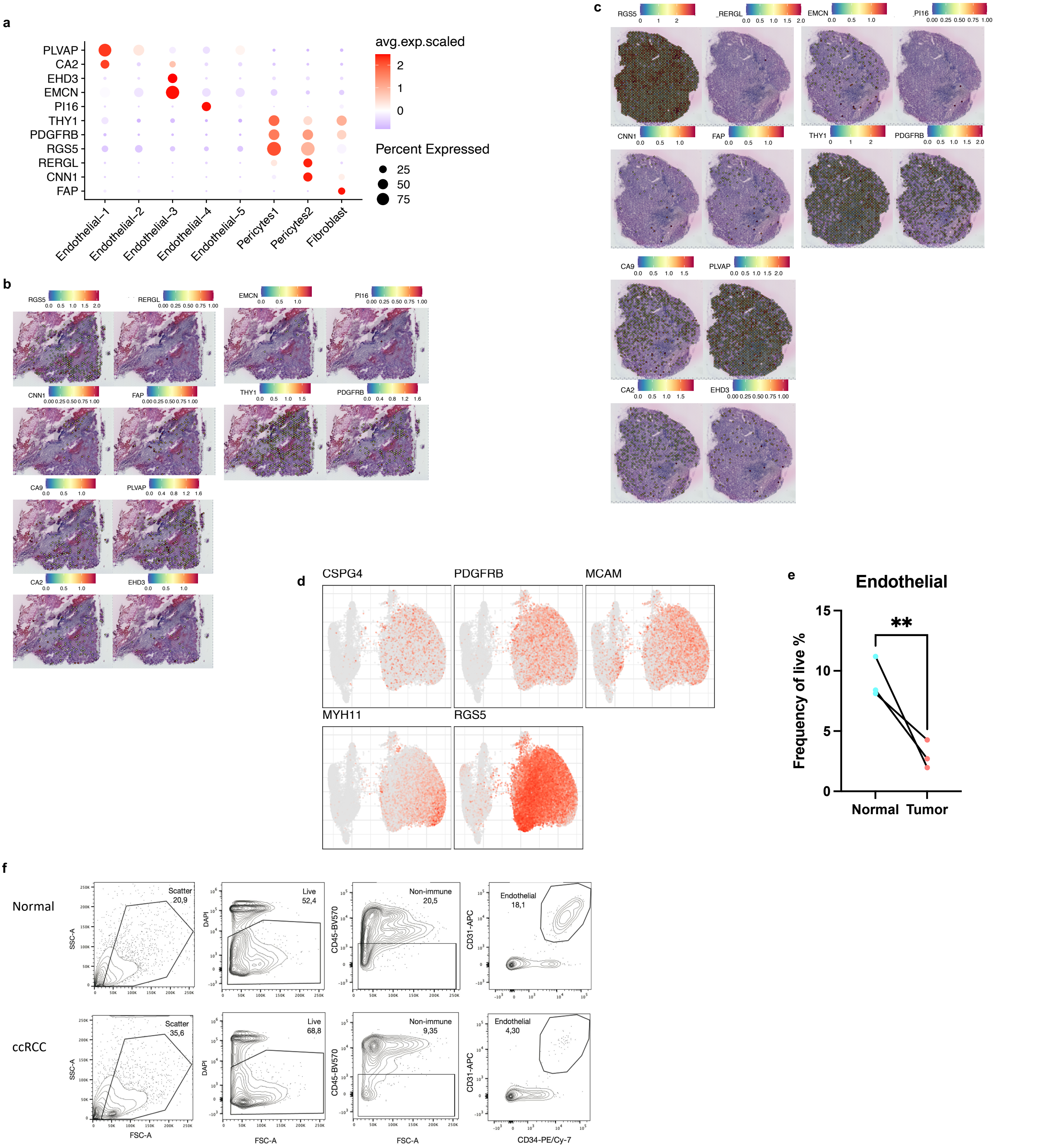

Supplementary Fig. 7: Spatial expression of stromal subpopulations in primary ccRCC. a Dotplot showing representative marker gene expression of stromal cell subpopulations in single cell data. **b-c** Spatial feature plots showing *CA9* and stromal cell marker gene expression in ccRCC patient. Tumor spots are marked by *CA9* expression. **d** Expression plot of common pericyte markers. **e** Plot of flow cytometry analysis of Endothelial (*CD34+, C31+*) visualized in frequency of live in ccRCC tumor and paired adjacent normal kidney tisse. Statistical analysis was performed by unpaired two tailed t-test (***p*=0,0063). **f** Flow cytometry gating strategy for endothelial population. Source data are provided as a Source Data file.

Supplementary Figure 8

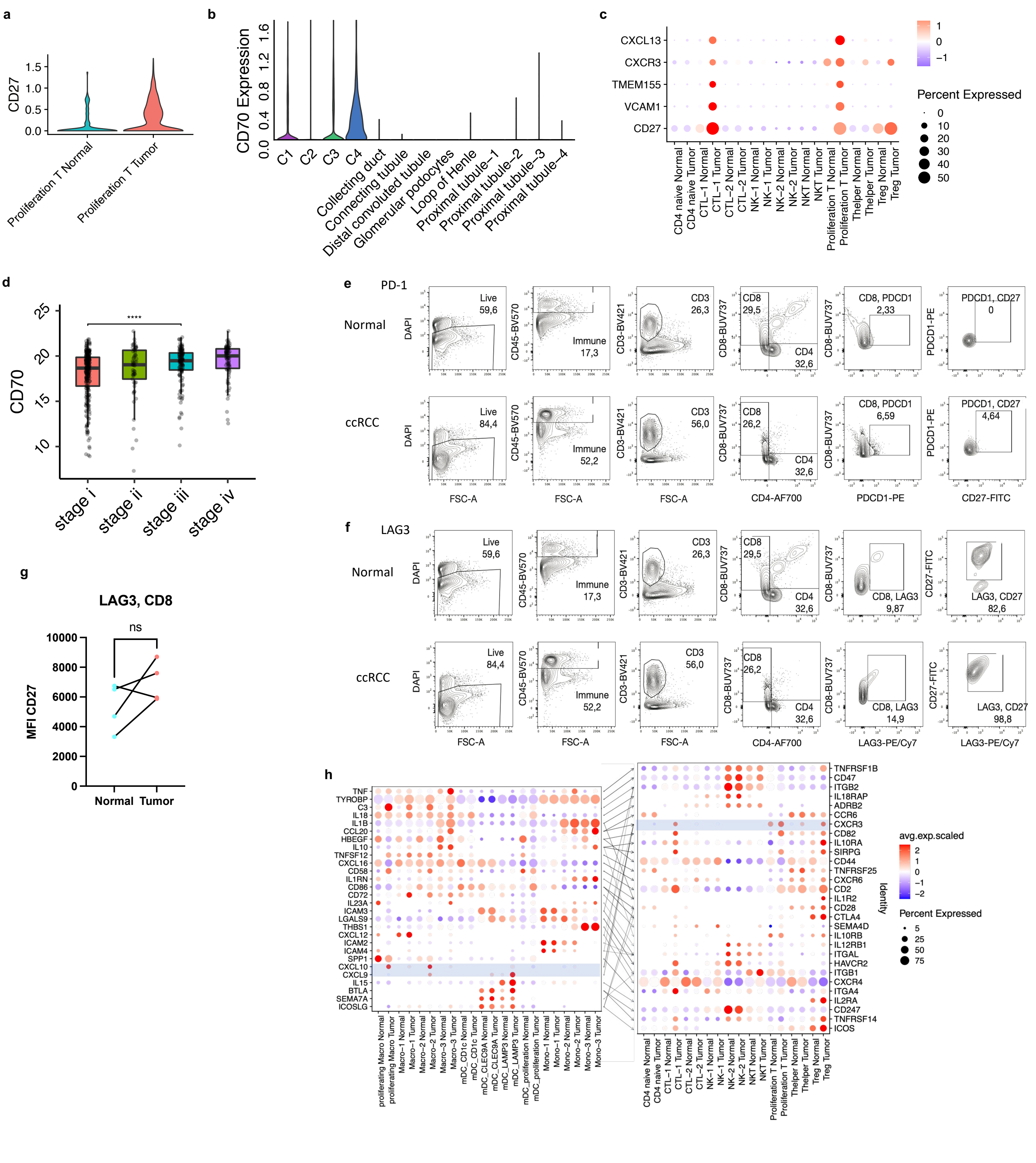

Supplementary Fig. 8: Ligand receptor analysis reveals CD70-CD27 mediated T cell exhaustion. a Violin plot showing *CD27* expression in proliferating T cells. **b** Violin plot showing *CD70* expression in malignant cell sub-clones and normal kidney tissue. **c** Dot plots showing representative marker genes across the different immune cell subsets. The colour represents scaled average expression of marker genes in each cell type, and the size indicates the proportion of cells expressing marker genes. **d** Expression of *CD70* is shown as boxplot, stratifying patients by disease stage (TCGA KIRC). Expression of *CD70* is shown as boxplot, stratifying patients by disease stage (TCGA KIRC) (stage i-iii ****p=6.14e-05; stage i-iv ****p=1.81e-07; ii-iv *p=0.042; iii-iv *p=0-048). Sample size: stage i= 267; stage ii=58, stage iii= 123; stage iv= 82. Boxplots include centreline, median; box limits, upper and lower quartiles; and whiskers are highest and lowest values no greater than 1.5x interquartile range. **e-f** Gating strategy for flow validation of *CD27* expression in *CD8+ PDCD+* T cells (**e**) and *CD8+ LAG3+* T cells (**f**). Labels above the flow plots refer to the parent population and the percentages are of parent gate. **g** Flow cytometric analysis of *CD27* expression on *CD8+ LAG3+* T cells from tumor and adjacent normal tissue. (n= 4 per group). Statistics are accessed with paired two tailed t-test (p = 0.1930, not significant). **h** Similar to **Figure 5B**, showing expression of ligand (myeloid cell subsets) and receptor (immune cell subsets) pairs in different myeloid and immune subsets. Source data are provided as a Source Data file.

Supplementary Figure 9

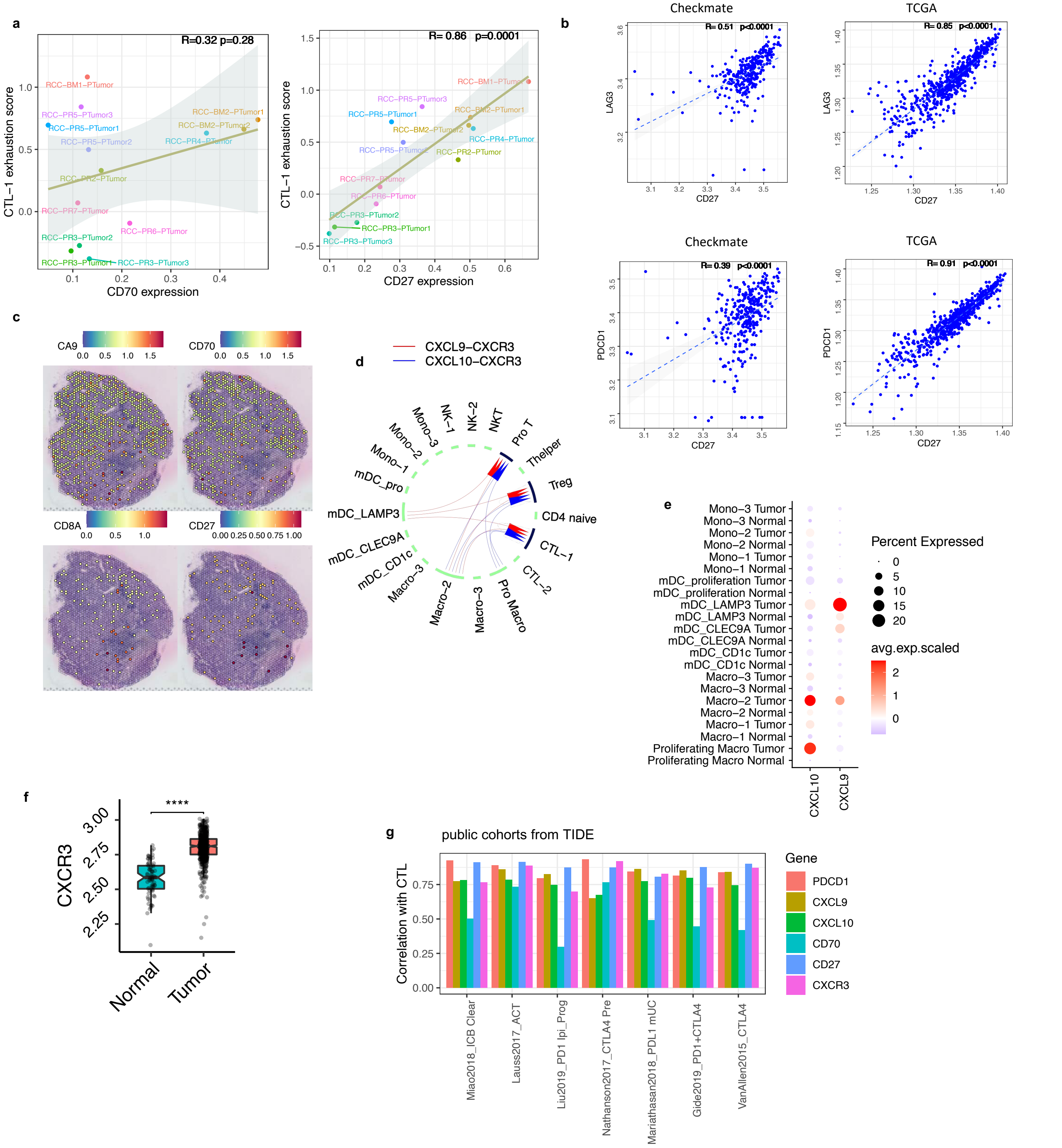

Supplementary Fig. 9: Ligand receptor analysis reveals potential therapeutic channels. a Similar to **Figure 5f**, showing correlation between *CD70* expression (tumor cells) and CTL-1 exhaustion score (left), correlation between *CD27* (CTL-1) expression CTL-1 exhaustion score (right). Pearson linear correlation estimate and p-values are shown. The error band indicates 95% confidence interval. CTL-1 exhaustion – CD70 expression: R=0.32, *p*=0.28, CI=95%; CTL-1 exhaustion – CD27 expression: R=0.86, *p*=0.0001. **b** Correlation of *CD27* expression and *LAG3/PDCD1* expression sore is shown as scatter plot for CheckMate (left) and TCGA KIRC (right) ccRCC bulk RNAseq data. **c** Spatial feature plots showing *CD70* and *CD27* expression in ccRCC patient. Tumor spots are marked by *CA9* expression. **d** The predicted interactions between *CXCL9/CXCL10* and *CRCR3*. **e** Dotplot representing *CXCL9* and *CXCL10* expression in myeloid cell subpopulations. The colour represents scaled average expression of marker genes in each cell type, and the size indicates the proportion of cells expressing marker genes. **f** *CXCR3* expression in TCGA KIRC data are shown as boxplot. Statistics are accessed with a two side Wilcoxon rank sum test (****p<2e-16, Sample size: Tumor n=533, Normal n=53). Boxplots include centreline, median; box limits, upper and lower quartiles; and whiskers are highest and lowest values no greater than 1.5x interquartile range. **g** Barplot showing correlation of estimated CTL infiltration and *PDCD1/CXCL9/CXCL10/CD70/CD27/CRCR3* expression in multiple immunotherapy trial studies from TIDE (http://tide.dfci.harvard.edu/query/). Source data are provided as a Source Data file.
