# Supplementary material for: A transcriptional metastatic signature predicts survival in clear cell renal cell carcinoma
Source: Nat Commun. 2022 Sep 30;13:5747. doi: 10.1038/s41467-022-33375-w (PMC9525645; doi:10.1038/s41467-022-33375-w)
Supplement: Supplementary file 3 — Reporting Summary [file 41467_2022_33375_MOESM3_ESM.pdf]

nature portfolio

Corresponding author(s): Ninib Baryawn, Peter V. Kharchenk, David B. Sykes, Philip J. Saylor

Last updated by author(s): Jun 29, 2022

Reporting Summary

Nature Portfolio wishes to improve the reproducibility of the work that we publish. This form provides structure for consistency and transparency in reporting. For further information on Nature Portfolio policies, see our Editorial Policies and the Editorial Policy Checklist.

Statistics

For all statistical analyses, confirm that the following items are present in the figure legend, table legend, main text, or Methods section.

n/a	Confirmed	
☐	☒	The exact sample size (*n*) for each experimental group/condition, given as a discrete number and unit of measurement
☐	☒	A statement on whether measurements were taken from distinct samples or whether the same sample was measured repeatedly
☐	☒	The statistical test(s) used AND whether they are one- or two-sided<br>*Only common tests should be described solely by name; describe more complex techniques in the Methods section.*
☒	☐	A description of all covariates tested
☐	☒	A description of any assumptions or corrections, such as tests of normality and adjustment for multiple comparisons
☐	☒	A full description of the statistical parameters including central tendency (e.g. means) or other basic estimates (e.g. regression coefficient) AND variation (e.g. standard deviation) or associated estimates of uncertainty (e.g. confidence intervals)
☐	☒	For null hypothesis testing, the test statistic (e.g. *F*, *t*, *r*) with confidence intervals, effect sizes, degrees of freedom and *P* value noted<br>*Give P values as exact values whenever suitable.*
☒	☐	For Bayesian analysis, information on the choice of priors and Markov chain Monte Carlo settings
☒	☐	For hierarchical and complex designs, identification of the appropriate level for tests and full reporting of outcomes
☐	☒	Estimates of effect sizes (e.g. Cohen's *d*, Pearson's *r*), indicating how they were calculated

Our web collection on statistics for biologists contains articles on many of the points above.

Software and code

Policy information about availability of computer code

Data collection	NextSeq 500 Sequencing System (Illumina) and bcl2fastq 1.8.4 were used to acquire data.
Data analysis	The sequenced 10X libraries were mapped to GRCh38 human genome using Cell Ranger software (version 3.0.1), Conos were used to integrate multiple scRNA-seq datasets. inferCNV (version 1.3.3) was used to predict copy number variations (CNVs). We perform RNA velocity analysis using python package velocity (version 0.17) and scvelo (version 0.2.3). Seurat (v4.0.6) was used to regress out cell cycle genes. cacoa (v0.3.0) was used to estimate differential cell density and expression distance. We use DEseq2 (v 1.32.0) and to call differential expressed genes and GOstat (v.4.2) for GO analysis. Statistics significance testes and plots were generated with R (version 4.1.1). The custom code used in this study can be found in this link https://github.com/shenglinmei/ccRCC.analysis/. All other codes are available from the corresponding author upon request.

For manuscripts utilizing custom algorithms or software that are central to the research but not yet described in published literature, software must be made available to editors and reviewers. We strongly encourage code deposition in a community repository (e.g. GitHub). See the Nature Portfolio guidelines for submitting code & software for further information.

Data

Policy information about availability of data

All manuscripts must include a data availability statement. This statement should provide the following information, where applicable:

- Accession codes, unique identifiers, or web links for publicly available datasets
- A description of any restrictions on data availability
- For clinical datasets or third party data, please ensure that the statement adheres to our policy

Raw single-cell RNA sequencing data and processed data can be accessed from the NCBI Gene Expression Omnibus database (accession code GSE178481). GRCh38 human reference genome was download from 10X genomics (https://support.10xgenomics.com/single-cell-gene-expression/software/downloads/). For the joint alignment analysis with public ccRCC scRNA-seq data, we downloaded raw count matrix and cell annotation from European Genome-phenome Archive (EGA: EGAS00001002171, EGAS00001002486, EGAS00001002325) and Single Cell Portal (https://singlecell.broadinstitute.org/single_cell/study/SCP1288/). 10X Visium Spatial Transcriptomics (ST) data were download from GSE175540. For bulk RNAseq data, TCGA clear cell renal cell carcinoma (KIRC) cohort were downloaded from the cBioPortal (https://www.cbioportal.org/study/clinicalData?id=kirc_tcga). The Checkmate 025 cohort data were obtained from David et.al 2020, Nature medicine.

Human research participants

Policy information about studies involving human research participants and Sex and Gender in Research.

Reporting on sex and gender	All 10 patients were male, age range 52-77. Tumor T-stage ranged from pT1a to pT3a and two cases of bone metastatic ccRCC.
Population characteristics	All patients underwent partial or radical nephrectomy for known or suspected renal cell carcinoma. Demographics reflect local practice pattern/population and disease demographics. All participants were male with the ages between 52 and 77 years old, all of them are male patient. See Supplementary Data 1 for exact details.
Recruitment	Participants were recruited from within the urologic surgery practice at Massachusetts General Hospital in Boston. All participants were planned for surgery for standard clinical indications. Informed consent was obtained prior to surgery. The participants were recruited with the criteria if they had a known kidney tumor ranging from stages pT1a to PT3a to investigate the cancer type at the primary stages. Two ccRCC bone metastatic patients included where recruited from the Neurosurgery at Massachusetts General Hospital.
Ethics oversight	Mass General Brigham IRB oversight of protocol: MGH GU Oncology Tissue Bank (IRB Protocol No: 2003P000641). MGH Neurosurgery (IRB approval number: 2017P000635/PHS).<br>Approving institute: Dana Farber Cancer institute and Partners Human Research Committee.

Note that full information on the approval of the study protocol must also be provided in the manuscript.

Field-specific reporting

Please select the one below that is the best fit for your research. If you are not sure, read the appropriate sections before making your selection.

☒ Life sciences ☐ Behavioural & social sciences ☐ Ecological, evolutionary & environmental sciences

For a reference copy of the document with all sections, see nature.com/documents/nr-reporting-summary-flat.pdf

Life sciences study design

All studies must disclose on these points even when the disclosure is negative.

Sample size	157,881 single-cells coming from 26 samples were included in this study. Sample sizes were chosen in a manner commensurate with similar previous studies (Bi et al., 2021, and Brian et al., 2021).
Data exclusions	We applied two general quality measures on raw gene-cell-barcode matrix for each cell: Total UMI > 700 and Scrublet scores < 0.4.<br>One sample was excluded in this study which was a sample collection from a patient diagnosed with another type of RCC, papillary renal cell carcinoma. This sample represented a markedly different CNV profile which deviates from the main finding of our data which is focused on clear cell renal cell carcinoma and the metastatic signature that predicts survival rather than a comparison between the different RCC.
Replication	Biological replicates from multiple patients were included in our data. Replication within single cell RNA sequencing is defined as recurrent results in all patients. In this study we have 10 patients, hence 10 replicates of our results. We also confirm our methodological approach and findings with literature of papers that also used single cell RNA sequencing on human RCC patients published in Cell, (Bi et al., 2021, and Brian et al., 2021).
Randomization	The patients with ccRCC were recruited randomly in this study.

Blinding | Not applicable. We did not use treatment or any other form of exposure to the patients directly to measure any outcome.

Reporting for specific materials, systems and methods

We require information from authors about some types of materials, experimental systems and methods used in many studies. Here, indicate whether each material, system or method listed is relevant to your study. If you are not sure if a list item applies to your research, read the appropriate section before selecting a response.

Materials & experimental systems

n/a	Involved in the study
☐	☒ Antibodies
☒	☐ Eukaryotic cell lines
☒	☐ Palaeontology and archaeology
☒	☐ Animals and other organisms
☒	☐ Clinical data
☒	☐ Dual use research of concern

Methods

n/a	Involved in the study
☒	☐ ChIP-seq
☐	☒ Flow cytometry
☒	☐ MRI-based neuroimaging

Antibodies

Antibodies used	See Supplementary Data 8 for specific antibody information regarding dilution, catalog number etc.
Validation	Validation of antibodies of our specific target as TREM2 was performed via gating strategy shown in Extended Data 2i ensuring that the antibody is binding a macrophage population (CD45+ CD11b+ CD68+). Similarly, we ensured antibody binding for a certain cell population through gating strategy the stromal cells (CD45 negative cells).

Flow Cytometry

Plots

Confirm that:

☒ The axis labels state the marker and fluorochrome used (e.g. CD4-FITC).

☒ The axis scales are clearly visible. Include numbers along axes only for bottom left plot of group (a 'group' is an analysis of identical markers).

☒ All plots are contour plots with outliers or pseudocolor plots.

☒ A numerical value for number of cells or percentage (with statistics) is provided.

Methodology

Sample preparation	Samples were thawed and labeled with the antibodies for each panel that were going to be analyzed.
Instrument	BD FACS Aria III equipped with a 100um nozzle (BD Biosciences, San Jose, CA) instrument
Software	FlowJO version 10.8.1, Java 9.0.1+11 and GraphPad Prism v.9.3.1 (350)
Cell population abundance	No sorting was done.
Gating strategy	The gating strategy followed the same procedure for all samples:<br>1 - Gating for live dead. Noise was considered dots that were not abundant within a clear population.<br>2- FSC-W (y axis) and FSC-A (x-axis) of singlets to correct for the granularity of the cell abundance<br>3- SSC-W (y axis) and SSC-A (x-axis) of singlets to correct for the size of the cell abundance<br>4 - Live/dead gating (y-axis) and FSC-A (x-axis). Cell population negative for the live/dead stain was proceeded for further analysis.<br>5a - For stromal populations: CD45 negative were selected with a FSC-A for further analysis<br>5b - For myeloid and T cell population: CD45 positive were selected with a FSC-A for further analysis<br><br>We utilize FMO strategy to confirm tha gating strategy for each desired population. Hence FMO was used throughout all panels that were analyzed and for each marker applied.

☒ Tick this box to confirm that a figure exemplifying the gating strategy is provided in the Supplementary Information.
